# Behavioral response to fluoxetine in both female and male mice is modulated by dentate gyrus granule cell activity

**DOI:** 10.1016/j.ynstr.2020.100257

**Published:** 2020-10-17

**Authors:** Christine N. Yohn, Andrew Dieterich, Isabella Maita, Allyson S. Bazer, Emma Diethorn, Debbie Ma, Mark M. Gergues, Pu Hu, Benjamin A. Samuels

**Affiliations:** a Behavioral & Systems Neuroscience, Department of Psychology, USA; b Neuroscience Graduate Program, Rutgers, The State University of New Jersey, 152 Frelinghuysen Rd, Piscataway, NJ, USA

**Keywords:** Antidepressant, Dentate gyrus, Sex differences, Chronic stress, Depression, Anxiety

## Abstract

Depression is a complex psychiatric disorder that is a major burden on society, with only ~33% of depressed patients attaining remission upon initial monotherapy with a selective serotonin reuptake inhibitor (SSRI). In preclinical studies using rodents, chronic stress paradigms, such as chronic corticosterone and social instability stress, are used to induce avoidance behaviors associated with negative affective states. Chronic fluoxetine (FLX; an SSRI) treatment reverses these chronic stress-induced behavioral changes in some, but not all mice, permitting stratification of mice into behavioral responders and non-responders to FLX. We previously reported that 5-HT_1A_ receptors, which are Gi-coupled inhibitory receptors, on mature granule cells (GCs) in the dentate gyrus (DG) are necessary and sufficient for the behavioral, neurogenic, and neuroendocrine response to chronic SSRI treatment. Since inhibition of mature DG GCs through cell autonomous Gi-coupled receptors is critical for mounting an antidepressant response, we assessed the relationship between behavioral response to FLX and DG GC activation in FLX responders, non-responders, and stress controls in both male and female mice. Intriguingly, using disparate stress paradigms, we found that male and female behavioral FLX responders show decreased DG GC activation (as measured by cFos immunostaining) relative to non-responders and stress controls. We then show in both sexes that chronic inhibition of ventral DG GCs (through usage of Gi-DREADDs) results in a decrease in maladaptive avoidance behaviors, while ventral DG GCs stimulation with Gq-DREADDs increases maladaptive behaviors. Finally, we were able to bidirectionally control the behavioral response to FLX through modulation of DG GCs. Chronic inhibition of ventral DG GCs with Gi-DREADDs converted FLX non-responders into responders, while activation of ventral DG GCs with Gq-DREADDs converted FLX responders into non-responders. This study illustrates ventral DG GC activity is a major modulator of the behavioral response to FLX in both male and female mice.

## 1 Introduction

Mood disorders, such as depression, are increasingly prevalent in society. Nearly 16% of Americans experience an episode of major depression in their lifetime and over 300 million people are affected worldwide (Kessler et al., 2003; WHO 2017). Stressful life events are risk factors for the onset of mood disorders, and patients show decreased levels of neuroplasticity, structural changes due to increased glucocorticoid levels, and decreased expression of neurotrophic factors. Antidepressant treatment enhances neuroplasticity and counteracts the impact of chronic stress (Calabrese et al., 2009; Joels 2011; Pittenger and Duman 2008). The most prescribed class of antidepressants are selective serotonin reuptake inhibitors (SSRIs), which ultimately lead to an increase in available serotonin (5-HT) levels by blocking reuptake into serotonergic neurons. Despite the widespread usage of SSRIs, 2 out of 3 patients with major depressive disorder do not remit after initial antidepressant monotherapy (Rush et al., 2006). Therefore, understanding the physiology of the antidepressant response and treatment resistance will lead to more effective treatments and therapies.

The dentate gyrus (DG), a subregion of the hippocampus, is strongly implicated in mediating the antidepressant response. Fourteen distinct 5-HT receptors exist, but 5-HT1A receptors, which are Gi-coupled heteroreceptors, on DG granule cells (GCs) are specifically required for the antidepressant response (Samuels et al., 2015; Yohn et al., 2017). Mice lacking 5-HT1A receptors on mature DG GCs show no behavioral response to SSRIs. Furthermore, 5-HT1A-receptor-deficient mice engineered to transgenically express 5-HT1A receptors on DG GCs show a behavioral response to SSRIs (Samuels et al., 2015). The ventral DG (vDG) is highly enriched in 5-HT1A receptors and regulates avoidance behaviors. Acute optogenetic inhibition of the ventral hippocampus suppresses innate avoidance behaviors in tasks such as the open field (OF) (Bagot et al., 2015), potentially mimicking an antidepressant response. Furthermore, chemogenetic inhibition of the vDG promotes resilience to chronic stress, whereas excitation promotes susceptibility (Anacker et al., 2018). Within the DG, chronic antidepressant treatment also increases all stages of adult neurogenesis, including proliferation of neural precursor cells, differentiation into young adult-born granule cells (abGCs), rate of maturation of young abGCs into mature DG GCs and integration of DG GCs into existing neural circuitry (Wang et al., 2008). Ablation of adult neurogenesis in the hippocampus via x-irradiation blocks the behavioral effects of antidepressants, suggesting that adult hippocampal neurogenesis is also necessary for the antidepressant response (David et al., 2009; Malberg et al., 2000; Santarelli et al., 2003; Wang et al., 2008; Yohn et al., 2017). Interestingly, increased neurogenesis promotes resilience to chronic stress and decreased activity in mature DG GCs, indicating that DG abGCs can inhibit mature GCs (Anacker et al., 2018). Specifically, entorhinal cortex projections to DG exert bidirectional effects on mature DG GCs activity via abGCs. DG abGCs activated via inputs from the lateral entorhinal cortex inhibit mature GCs through metabotropic glutamate receptors, while medial entorhinal cortex projections into the DG lead to abGCs exciting mature GCs through NMDA receptors (Luna et al., 2019).

Novelty suppressed feeding (NSF) can assess behavioral responder/non-responder patterns to antidepressant treatment (Gergues et al., 2018; Samuels et al. 2011, 2014). In NSF, latency to approach and eat a food pellet in the center of a brightly lit arena after 18–24 h of food deprivation is observed. A longer latency to eat suggests either increased maladaptive avoidance behavior or decreased motivation to take risks for food rewards. Chronic SSRI treatment significantly reduces latency to eat in mice. However, latency to eat data are often bimodally distributed with a subset of antidepressant-treated mice retaining a long latency to eat (Gergues et al., 2018; Samuels et al. 2011, 2014). This bimodal distribution permits stratification of mice into presumptive responders (shorter latency to eat) and non-responders (longer latency to eat) to SSRI treatment. Here we assess the role of the DG in the behavioral antidepressant response. Inhibition of mature DG GCs, via activation of Gi-coupled 5-HT_1A_ receptors, is essential for the antidepressant response (Samuels et al., 2015). Therefore, by utilizing chemogenetic DREADD-mediated inhibition and excitation, we seek to clarify the role of the vDG in the antidepressant response. We predict that Gi-DREADD-mediated inhibition of vDG GCs will mimic an antidepressant response, while Gq-DREADD-mediated activation of vDG GCs will increase maladaptive avoidance behaviors. We also aim to understand if Gi-DREADD-mediated silencing of DG GCs can convert FLX non-responders into responders.

## 2 Materials and methods

### 2.1 Subjects

Adult male and female C57BL/6 J mice (age 8 weeks) were purchased from Jackson Labs (Bar Harbor, ME). Mice were maintained on a 12 L:12D schedule with lights turned on at 6 a.m. and lights turned off at 6pm. Food and water were provided ad libitum. All behavioral testing occurred in the light phase between the hours of 8 a.m. and 11 a.m. All testing was conducted in compliance with the NIH lab animal care guidelines and was approved by the Rutgers University Institution of Animal Care and Use Committee.

### 2.2 Stress paradigms

#### 2.2.1 Chronic corticosterone

Adult male C57BL/6 J mice were randomly divided into Corticosterone (CORT) and vehicle (VEH) treatments, with weights measured on a weekly basis during treatment. VEH treated mice were administered 0.45% beta-cyclodextrin dissolved in their drinking water, whereas CORT treated mice received a CORT (35 μg/mL) (Sigma-Aldrich, St. Louis, MO) dissolved in 0.45% beta-cyclodextrin (4.5 mg/mL) (Sigma-Aldrich, St. Louis, MO). CORT was administered in opaque bottles due to the light sensitivity of the drug (David et al., 2009). CORT treatment lasted the duration of all experiments it was involved in.

#### 2.2.2 Social instability stress (SIS)

Female C57BL/6 J mice were randomly assigned into SIS or control (CNTRL) groups. SIS mice were subject to fluctuating and unstable social environments in which social dynamics are changed twice a week for 7 weeks (Yohn et al., 2019). Specifically, cage composition was changed twice a week so that mice were housed with novel mice of the same sex. The total number of mice per cage at any given time ranged from 3 to 5 mice. The rotation schedule was randomized to prevent mice from being housed with a recent house mate. Following the initial 7-week exposure to SIS, SIS continued for the duration of the experiments. Cage composition changes did not occur on behavioral testing days. Female mice in the CNTRL group were housed with the same mice during the entire duration of the SIS paradigm and cages were changed twice per week.

### 2.3 Viral injections

To assess antidepressant-like effects of inactivation or activation of vDG GCs one of three DREADD viruses pAAV8-CamKIIa-hm4D(Gi)-mCherry (Addgene V7857), pAAV8-CamKIIa-hm3D(Gq)-mCherry (Addgene V4490), pAAV8-CamKIIa-EGFP-mCherry (Addgene V4489) were injected bilaterally into the vDG of 6-week-old mice: −3.5 mm, ±2.8 mm relative to the bregma line and midline respectively at a depth of −3.6 mm from the skull at the bregma (Fig. 3a). Using a nanoinject III (Drummond Scientific), one of the three viruses was delivered to the target sight, with a total volume of 300 nL at a flow rate of 1–2 nL per second.

### 2.4 Drug treatment

#### 2.4.1 Fluoxetine (FLX)

Fluoxetine hydrochloride (18 mg/kg/day in deionized water; Biotrend BG0197) or VEH (deionized water) was administered via oral gavage for three weeks prior to behavioral testing. Oral gavaging of FLX or VEH solution continued through behavioral testing. During behavioral testing days, oral gavage was delivered after behavior to avoid any acute effects.

#### 2.4.2 Clozapine n-oxide (CNO)

Water bottles were filled on alternating days with 0.05 mg/ml CNO (hello bio). CNO was dissolved in dimethyl sulfoxide (DMSO, Fisher Scientific), for a final concentration of 0.25% DMSO, and added to 0.5% saccharine water. Mice weighed between 30 and 35 g and water consumption was approximately 3 ml/day, so each mouse received an approximate oral dose of 5 mg/kg CNO daily (Zhan et al., 2019). CNO treatment was given chronically for 3 weeks in both experiments it was involved in (Fig. 3, Fig. 4).

### 2.5 Behavioral testing

#### 2.5.1 Open field (OF)

To assess motor activity, mice were placed in the corner of a Plexiglas open field chamber measuring 43 cm × 43 cm and monitored with Motor Monitor software (Kinder Scientific, Poway, CA). Through infrared photobeams on the wall, the software measured distance traveled (cm) and time in the periphery and center of the OF. The center was defined as a square 11 cm from each wall of the OF. We also calculated percent distance traveled ((center distance/total distance)*100) in our analyses.

#### 2.5.2 Novelty suppressed feeding (NSF)

After an 18-h food deprivation period, mice were placed in the corner of a novel, brightly lit NSF chamber that contained a food pellet in the center. The mice were observed and latency to eat the food pellet was recorded.

#### 2.5.3 Forced swim test (FST)

Mice were placed in a Forced Swim Test chamber filled with room temperature water for 6 min. Motor Monitor software (Kinder Scientific, Loway, CA) was used to measure immobility, defined as 6 or less beam breaks over the span of 5 s, in the last 4 min of the test.

#### 2.5.4 Light dark (LD)

A dark rectangular box opaque to visible light and containing a small opening permitting mice to move between light (1000 lux) and dark compartments covered ⅓ of the OF arena. Mice were placed in the dark compartment of the arena and movements were measured (Motor Monitor, Kinder Scientific). Percent distance traveled in the light compartment (distance traveled in the light compartment/total distance * 100) was used for analysis.

#### 2.5.5 Elevated plus maze (EPM)

The elevated plus maze is a plus shaped apparatus raised 2 feet above floor level that consists of two closed and two open arms. At the start of the 5-min test, mice are placed in the center of the plus maze and video recorded by a camera mounted on the ceiling above the EPM. Using EthoVision software (Noldus, Wageningen, Netherlands) time spent in the open arms and distance traveled in both open and closed arms were assessed. In our analysis, the percent distance traveled was derived ((total open arm distance traveled/total distance traveled)*100).

### **2.6** cFos immunohistochemistry (IHC)

The effects of chronic stress (CORT or SIS) in the presence or absence of fluoxetine treatment on DG GC activity was assessed through cFos analysis (timelines Fig. 2a and e). We also used cFos to confirm inhibition or excitation of the vDG in DREADD experiments (Fig. 3a and i) (Zhan et al., 2019). Forty minutes after NSF task exposure, animals were anesthetized with ketamine and xylazine (100 mg/ml ketamine; Henry Schein, Melville, NY; 20 mg/ml xylazine; Sigma Aldrich, St. Louis, MO), and perfused transcardially (cold saline for 2 min, followed by 4% cold paraformaldehyde at 4 °C). The brains were removed and stored in 4% paraformaldehyde (Sigma Aldrich) overnight at 4 °C. Next, brains were cryoprotected in 30% sucrose (ThermoFisher), 1% sodium azide (Sigma Aldrich), and stored at 4 °C. Serial sections (40 μM) were cut on a cryostat and collected through the entire hippocampus (Franklin, 1997). Sections were collected in wells and wet mounted prior to staining. Sections were washed in 1% Triton X- 100 (Millipore Sigma, Burlington, MA) Phosphate Buffer solution (PBS; Millipore Sigma) for 5 min before undergoing three PBS washes. Slides were incubated in warm citrate buffer for 30 min. After washing with PBS, slides were blocked for 1 h in 10% normal goat serum (NGS; MP Biomedicals, Solon, OH) before being incubated overnight at 4 °C in anti-rabbit cFos (1:750; Cell Signal Technology, 9F6). Next, slides were washed with PBS then incubated at room temperature for 2 h in the secondary antibody (CY-5 goat anti-rabbit; 1:1000, Abcam or FITC goat anti-rabbit, 1:1000, Invitrogen). Following the incubation, slides were washed with PBS then counterstained with DAPI (1:15000; Invitrogen) for 15 min. Finally, slides were washed with PBS and cover slipped using the mounting medium prolong diamond (Thermo Fisher). Fluorescent images were taken using an inverted microscope (ThermoFisher), where cFos positive cells overlaid with DAPI across the 12 sections of hippocampus will be counted.

## 3 Results

### 3.1 Responder and non-responder phenotypes to FLX in both male and female mice

**Fig. 1:**
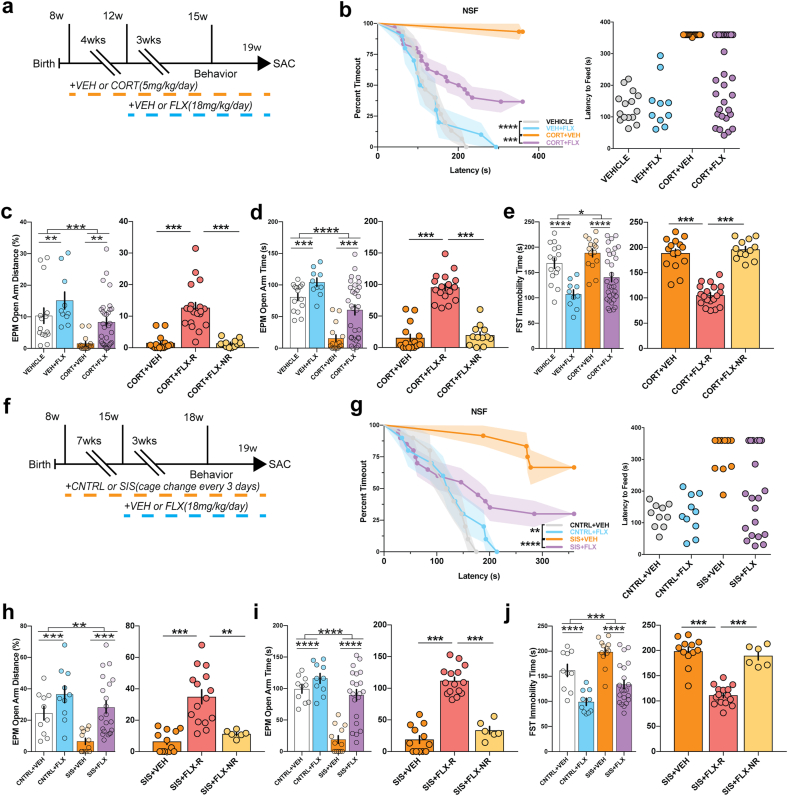
Male and female behavioral responders and non-responders to chronic fluoxetine treatment. Timeline of experiment males (a) and females (f). (b & g) NSF was used to distinguish responders and non-responders to chronic FLX treatment. Kaplan-Meier Survival Analysis with Bonferroni corrections. (left graphs c-e; h-j) 2 × 2 ANOVAs were conducted to examine stress and treatment effects, and then separate one-way ANOVAs (right graphs c-e; h-j to investigate differences between stress only, stress + FLX responders, and stress + FLX non-responders. **p < 0.01; ***p < 0.001, ****p < 0.0001. (sample sizes males: VEH + VEH = 15, VEH + FLX = 10, CORT + VEH = 15, CORT + FLX = 31 (CORT + FLX-R = 19, CORT + FLX-NR = 12); females: CNTRL + VEH = 10, CNTRL + FLX = 10, SIS + VEH = 12, SIS + FLX = 21 (SIS + FLX-R = 15, SIS + FLX-NR = 6).

To investigate antidepressant treatment resistance following chronic stress, we exposed a cohort of group housed 8-week-old male C57BL/6 J mice to chronic vehicle (VEH) or corticosterone (CORT, 5 mg/kg/day via drinking water) administration. Chronic CORT administration at this dosage induces several maladaptive avoidance behaviors, including increased latency to feed in NSF and decreased open arm entries and duration in the elevated plus maze (EPM) (David et al., 2009). We administered vehicle or CORT for 4 weeks and then co-administered either VEH or the SSRI fluoxetine (FLX, Prozac, 18 mg/kg/day) for an additional 3 weeks (timeline Fig. 1a). As expected, we found group differences in latency to feed in the NSF (x(3) = 36.75, p < 0.000001, logrank Mantel-Cox test; Fig. 1b), with coadministration of CORT + FLX resulting in significantly reduced latency to feed relative to CORT + VEH treated mice (p = 0.00045, logrank Mantel-Cox with Bonferroni correction) and VEH + VEH mice (p < 0.000001, logrank Mantel-Cox with Bonferroni correction) (Fig. 1b). Similar to our previous findings (Gergues et al., 2018; Samuels et al. 2011, 2014), the individual latencies of CORT + FLX mice demonstrated a distribution that was not normal (Shapiro-Wilk normality test p < 0.0001) but rather appeared bimodal, providing a potential basis for dividing mice into responder and non-responder to FLX treatment groups. We designated mice that did not eat in the 6-min NSF as non-responders (assigned a time of 360 s in NSF), and all mice that did eat as responders. Food consumption in the home cage was similar among all mice (Supplemental Fig. 1a). We previously reported that serum levels of FLX metabolites are similar between NSF-defined responders and non-responders to CORT + FLX (Samuels et al., 2014).

**Fig. 2:**
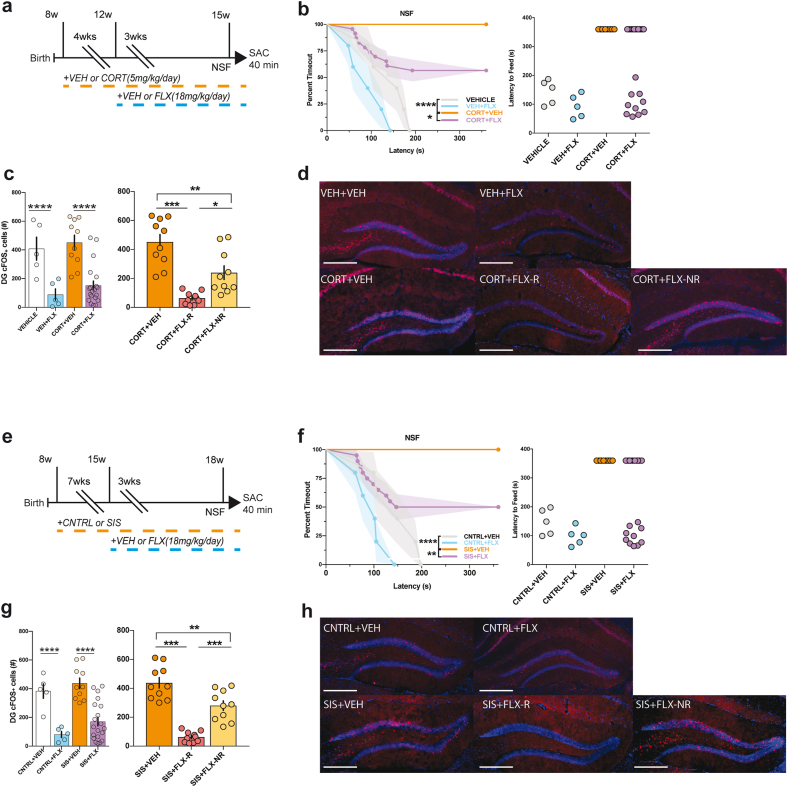
Differences in DG cFos expression between responders and non-responders to fluoxetine treatment. (a) Male and (e) female experimental timelines. (b & f) NSF was used to distinguish responders and non-responders to chronic FLX treatment. Kaplan-Meier Survival Analysis with Bonferroni corrections. (c & g) Mice were perfused to assess DG GC activity (as measured by cFos IHC) in response to NSF exposure. Both male (c) and female (g) responders had less DG cFos + cells than their stress and non-responder counterparts. (d & h) Representative cFos images (10x). **p < 0.01; ***p < 0.001, ****p < 0.0001. (sample sizes males: sample sizes males: VEH + VEH = 5, VEH + FLX = 5, CORT + VEH = 10, CORT + FLX = 20 (CORT + FLX-R = 10, CORT + FLX-NR = 10); females: CNTRL + VEH = 5, CNTRL + FLX = 5, SIS + VEH = 10, SIS + FLX = 20 (SIS + FLX-R = 10, SIS + FLX-NR = 10).

**Fig. 3:**
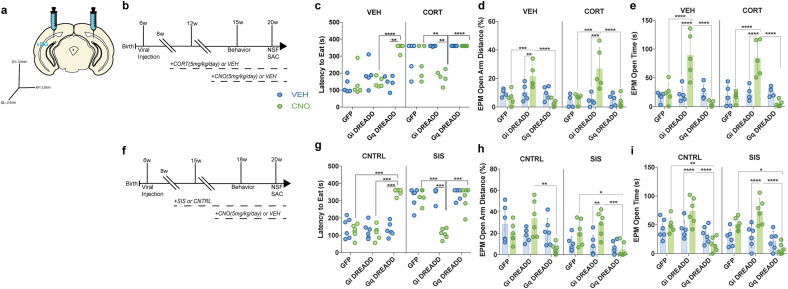
Opposing effects of vDG Gi-vs Gq-DREADD activation on avoidance behaviors in males and females. (a) Surgical representation of coordinates used to target the vDG. (b) Experimental timeline for males. (c) Gi DREADD + CNO mice had a shorter latency to eat than Gq DREADD + CNO in the NSF. (d–e) Gi-DREADD + CNO mice on either a VEH or CORT background traveled more and spent more time on the EPM open arms than GFP + CNO and Gq-DREADD + CNO treated mice. (f) Experimental timeline for females. (g) Stressed Gi DREADD + CNO mice had a shorter latency to eat than Gq DREADD + CNO in the NSF. (h–i) Gi-DREADD + CNO mice traveled more and spent more time on the EPM open arms than GFP + CNO and Gq-DREADD + CNO treated mice. **p < 0.01; ***p < 0.001, ****p < 0.0001. (sample sizes males: all groups n = 5; females: all groups n = 6).

**Fig. 4:**
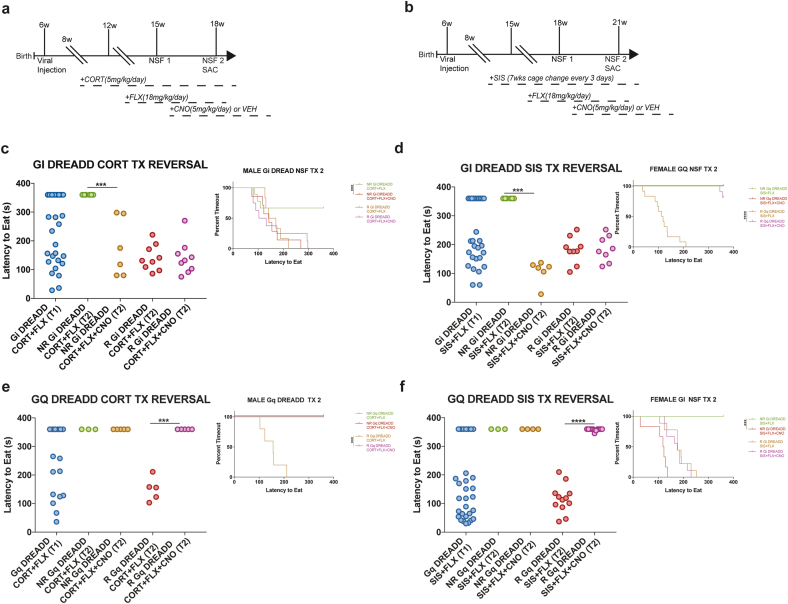
DREADD-mediated bidirectional control of behavioral fluoxetine response in both males and females. (a & b) Experimental timelines. (c & d) NSF latencies for Gi DREADD, with FLX non-responders (NR) showing a shorter latency to feed following vDG inhibition, indicating conversion into responders. (e & f) Chronic vDG stimulation via Gq-DREADD resulted in increased latency to feed in stimulated FLX responders compared to vDG unstimulated FLX responders, indicating conversion into non-responders. **p < 0.01; ***p < 0.001, ****p < 0.0001. (sample sizes males: Gi-DREADD (Gi-DREADD CORT + FLX-NR = 5, Gi-DREADD CORT + FLX-R = 6, Gi-DREADD CORT + FLX-NR + CNO = 5, Gi-DREADD CORT + FLX-R + CNO = 6), Gq-DREADD (Gq-DREADD CORT + FLX-NR = 3, Gq-DREADD CORT + FLX-R = 5, Gq-DREADD CORT + FLX-NR + CNO = 5, Gq-DREADD CORT + FLX-R + CNO = 5); females: Gi-DREADD (Gi-DREADD SIS + FLX-NR = 5, Gi-DREADD SIS + FLX-R = 9, Gi-DREADD SIS + FLX-NR + CNO = 5, Gi-DREADD SIS + FLX-R + CNO = 9), Gq-DREADD (Gq-DREADD SIS + FLX-NR = 3, Gq-DREADD SIS + FLX-R = 5, Gq-DREADD SIS + FLX-NR + CNO = 5, Gq-DREADD SIS + FLX-R + CNO = 5).

We next exposed the same cohort of C57BL/6 J mice to elevated plus maze (EPM), which assesses avoidance behavior, and the forced swim test (FST), which is a commonly used test of antidepressant efficacy. Two-way ANOVAs revealed effects of CORT administration and FLX treatment in EPM distance traveled on the open arms (CORT: F(1,67) = 16.12, p = 0.0002, FLX: F(1,67) = 8.83, p = 0.0041; Fig. 1c left), EPM open arm duration (CORT: F(1,67) = 40.3, p < 0.0001, FLX: F(1,67) = 15.81, p = 0.0002; Fig. 1d left), and FST immobility (CORT: (F(1,67) = 6.44, p = 0.013, FLX: F(1,67) = 26.68, p < 0.0001; Fig. 1e left). To investigate behavioral differences between CORT-only treated mice, NSF-defined CORT + FLX responders, and CORT + FLX non-responders in the EPM and FST, we used one-way ANOVAs and found significant differences in open arm distance traveled (F(2,43) = 30.03, p < 0.001; Fig. 1c right), open arm duration (F(2,43) = 73.71, p < 0.001; Fig. 1d right), and immobility F(2,43) = 72.13, p < 0.001; Fig. 1e right). Bonferroni-corrected post hoc tests demonstrated that responders had significantly increased open arm exploration (Fig. 1c right) and duration (Fig. 1d right), as well as decreased immobility (Fig. 1e right), relative to VEH treated mice and non-responders (CORT + VEH vs CORT + FLX-R and CORT + FLX-R vs CORT + FLX-NR, all p < 0.001). Taken together, these data suggest that FLX response status across NSF, EPM, and FST is conserved in CORT-treated male mice.

We next wanted to examine whether antidepressant response status is conserved across NSF, EPM, and FST in female mice. However, males and females are differentially affected by chronic stress paradigms. For example, CORT administration is far more effective in male mice (Mekiri et al., 2017; Yohn et al., 2019). We recently developed a chronic social stress paradigm, social instability stress (SIS), that induces maladaptive behaviors in female mice through exposure to unstable social environments by changing cage compositions every 3 days for at least 7 weeks (Yohn et al., 2019). To this end, we exposed a cohort of group housed 8-week-old female C57BL/6 J mice to chronic SIS or control (CNTRL). Following the initial 7 weeks of SIS, we administered VEH or FLX for an additional 3 weeks, while continuing SIS (Fig. 1f). Similar to CORT-treated males, we found significant group differences in latency to feed in the NSF (x(3) = 22.62, p = 0.000048, logrank Mantel-Cox test; Fig. 1g), with SIS + FLX treated mice having a significantly reduced latency to feed relative to SIS + VEH mice (p = 0.018), indicative of an antidepressant response. CNTRL + VEH mice showed a significant reduced latency to feed relative to SIS + VEH mice (p < 0.000001). Furthermore, the individual latencies of SIS + FLX females did not display a normal distribution (Shapiro-Wilk normality test p = 0.0002), instead appearing to be bimodal. Food consumption in the home cage was similar among all mice (Supplemental Fig. 1c).

We next exposed the same cohort of C57BL/6 J female mice to EPM and FST. Two-way ANOVAs revealed effects of SIS and FLX treatment in open arm distance (SIS: F(1,49) = 8.94, p = 0.0043, FLX: F(1,49) = 14.73, p = 0.0004) (Fig. 1h left), open arm duration (SIS: F(1,49) = 34.9, p < 0.0001, FLX: F(1,49) = 23.39, p < 0.0001) (Fig. 1i left), and immobility (SIS: F(1,49) = 12.53, p = 0.0009, FLX: F(1,49) = 40.15, p < 0.0001) (Fig. 1j left). NSF-defined responder and non-responder status was consistent across NSF, EPM, and FST, as one-way ANOVAs found significant differences in open arm distance (F(2,30) = 18.99, p < 0.001) (Fig. 1h right), open arm duration (F(2,30) = 74.2, p < 0.001) (Fig. 1i right), and immobility (F(2,30) = 55.8, p < 0.001) (Fig. 1j right), with responders having significantly increased open arm exploration and duration, and decreased immobility, relative to vehicle treated mice and non-responders (SIS + VEH vs SIS + FLX-R and SIS + FLX-R vs SIS + FLX-NR, p ≤ 0.002 for all). These data demonstrate that in different sexes, which were exposed to completely disparate stressors, FLX behavioral response status is conserved across behaviors.

### 3.2 Male and female behavioral responders to FLX show decreased DG GC activation during NSF

To assess the role of the DG in facilitating the behavioral response to antidepressants, we next treated separate cohorts of male and female mice with 4 weeks of CORT or VEH administration and SIS or CNTRL exposure, respectively. We then administered either VEH or FLX for 3 additional weeks (timelines: Fig. 2a & g). Following chronic FLX or VEH treatment, mice underwent NSF and were then sacrificed 40 min post-NSF to investigate expression of the immediate early gene cFos in DG. In the NSF, we found significant group differences in latency to feed in NSF (males: x(3) = 22.85, p = 0.000043; females: (x(3) = 21.16, p = 0.000097) (Fig. 2b and f). Coadministration of CORT/SIS and FLX resulted in significantly reduced latency to feed relative to CORT/SIS + VEH treated mice (males: p = 0.018, females: p = 0.0099) and VEH + VEH treated mice (males and females: p < 0.0001) (Fig. 2b and f). There were no differences in home cage feeding (Supplemental Figs. 1b and 1d). We collected and stained 1 out of every 6 sections containing the DG (total of 12 sections counted) to assess cFos expression across the DG GC layer (Fig. 2d and h). Two-way ANOVAs revealed FLX treatment (males: F(1,36) = 33, p < 0.0001; females: F(1,36) = 40.07, p < 0.0001), but not chronic stress (CORT in males: F(1,36) = 0.96, p = 0.332; SIS in females: F(1,36) = 2.57, p = 0.117), significantly reduces cFos expression in the DG (Fig. 2c & g left). To investigate differences in cFos expression between CORT/SIS-only treated mice, NSF-defined CORT/SIS + FLX responders, and non-responders, we used one-way ANOVAs, which showed CORT/SIS + FLX-R mice have fewer DG cFos + cells than CORT/SIS + VEH (males and females: p < 0.001) and CORT/SIS + FLX-NR mice (males: p = 0.016, females: p < 0.001) (Fig. 2c & g right). Taken together, these data demonstrate across two disparate chronic stressors and both sexes that NSF exposure results in less activated DG GCs in responders than in non-responders to FLX treatment.

### 3.3 Modulation of ventral DG with DREADDs mimics antidepressant responder/non-responder behavioral phenotypes

Based on the cFos data, and the fact that inhibition of DG GCs is important for mediating the antidepressant response and resilience to chronic stress (Anacker et al., 2018; Samuels et al., 2015), we next wanted to investigate the effects of Gi- and Gq-DREADD expression and activation in DG GCs on behavior. We hypothesized that Gi-DREADD-mediated inhibition of DG GCs in mice exposed to chronic stress would mimic an antidepressant response and that Gq-DREADD-mediated activation of DG GCs in stress naïve mice would mimic the effects of chronic stress. To this end, we bilaterally injected either AAV8-CamKIIa-hm4D(Gi)-mCherry, AAV8-CamKIIa-hm3D(Gq)-mCherry, or AAV8-CamKIIa-GFP into the ventral DG (vDG) of 6-week-old mice (Fig. 3a). We then administered 8-week-old males with CORT or VEH and exposed 8-week-old females to either SIS or CNTRL. After 4 weeks of CORT treatment or 7 weeks of SIS exposure, we then co-administered either VEH or CNO (average consumption of 5 mg/ml CNO daily) in the drinking water (Fig. 3b & f). To confirm that CNO activated the DREADD viruses we measured vDG cFos expression 40 min after NSF exposure. Two-way ANOVAs between treatment (CNO or VEH) and viral groups (Gi-DREADD, Gq-DREADD, GFP) showed significant interactions (males: F(2,30) = 21.2, p < 0.001; females: F(2,30) = 27.87, p < 0.001) (Supplemental Figs. 2c and 2e). Gi-DREADD + CNO mice had less vDG cFos + cells compared to GFP + CNO, Gq-DREADD + CNO, and Gi-DREADD + VEH (males and females: all p < 0.001). By contrast, Gq-DREADD + CNO mice had more vDG cFos expression than GFP + CNO (males: p = 0.040, females: p = 0.039) and Gq-DREADD + VEH (males: p = 0.046, females: p = 0.026) (Supplemental Figs. 2c and 2d). CNO had no effects relative to VEH in Control (AAV8-CamKIIa-GFP) injected mice. There were also similar levels of viral-mediated expression across all mice (Supplemental Figs. 2d and 2f). These results suggest that DREADD activation had the expected effects, with CNO administration leading to vDG inhibition in Gi-DREADD mice and to vDG activation in Gq-DREADD mice.

We next investigated behavioral effects of DREADD-mediated vDG activation or inhibition in NSF and EPM. To simplify the experiment, we ran separate analyses within naïve and chronic stress-exposed mice. We found significant differences in NSF latency to eat within stress naïve groups (VEH males: x^2^(5) = 17.99, p = 0.003; CNTRL females: x2(5) = 20.89, p = 0.0008) (Fig. 3c and g). Stress naïve Gq-DREADD + CNO mice have a longer latency to eat than Gi-DREADD + CNO (males: p < 0.0001, females: p = 0.0005) and Gq-DREADD + VEH (males: p = 0.0015, females: p = 0.0007) (Fig. 3c & f left). In chronic stress-exposed mice, we also found significant differences in NSF latency to eat (CORT males: x^2^(5) = 23.58, p = 0.0003; SIS females: x^2^(5) = 34.01, p < 0.0001), with Gi-DREADD + CNO mice eating significantly faster than Gi-DREADD + VEH (males: p = 0.0035, females: p = 0.0007), Gq-DREADD + CNO (males: p < 0.0001, females: p = 0.0007), and GFP + CNO (males: p = 0.0025; females: p = 0.0007) mice (Fig. 3c & g right). CNO had no effects in NSF relative to VEH in Control (AAV8-CamKIIa-GFP) injected mice. There were also no differences between or within groups in home cage latency to eat (Supplemental Figs. 4a and 4d).

We next ran separate two-way ANOVAs within naïve and chronic stress-exposed mice to analyze EPM avoidance behaviors. Within stress naïve mice, we found significant interactions between CNO treatment and virus injected in open arm distance (males: F(2, 24) = 8.534, p = 0.0016; females: F(2, 30) = 6.817, p = 0.0036) and duration (males: F(2, 24) = 12.7, p = 0.0002; females: F(2, 30) = 5.34, p = 0.0103) (Fig. 3d–e & 3 h-i left). In males, VEH Gi-DREADD + CNO mice traveled more distance and spent more time on EPM open arms than VEH Gi-DREADD + VEH (distance: p = 0.0078; time: p < 0.0001), VEH Gq-DREADD + CNO (distance: p < 0.0001; time: p < 0.0001), and VEH GFP + CNO (distance: p = 0.0004; time: p < 0.0001) mice (Fig. 3d–e). In females, CNTRL Gq DREADD + CNO mice spent less time on the open arms than CNTRL Gi DREADD + CNO (p < 0.0001) and CNTRL GFP + CNO (p = 0.0041), and traveled less open arm distance than CNTRL Gi DREADD + CNO (p = 0.001). Also, CNTRL Gi DREADD + CNO female mice spent more time on the EPM open arms than Gi DREADD + VEH (p = 0.0053) (Fig. 3h–i left). Within chronic stress-exposed mice, we also found significant interactions between CNO treatment and virus injected in EPM open arm distance (males: F(2, 24) = 8.446, p = 0.0017; females: F(2,30) = 4.53,p = 0.019) and duration (males: F(2, 24) = 17.03, p < 0.0001; females: F(2,30) = 9.631,p = 0.0006 (Fig. 3d–e & 3 h-i right). CORT Gi DREADD + CNO male mice traveled more (Fig. 3d right) and spent more time (Fig. 3e right) on EPM open arms than CORT Gi-DREADD + VEH (distance: p = 0.0001; time: p < 0.0001), CORT Gq-DREADD + CNO (distance: p < 0.0001; time: p < 0.0001), and CORT GFP + CNO (distance: p = 0.0003; time: p < 0.0001) male mice (Fig. 3d–e right). SIS Gq DREADD + CNO mice traveled less distance and spent less time in the EPM open arms than SIS GFP + CNO (distance: p = 0.0123; time: p = 0.0002) and SIS Gi DREADD + CNO (distance: p = 0.0002; time: p < 0.0001). Also, SIS Gi DREADD + CNO mice traveled more distance and spent more time on the EPM open arms than SIS Gi DREADD + CNO (distance: p = 0.0014; time: p = 0.0002) (Fig. 3h–i right).

We also ran open field (OF) and light/dark emergence (LD) tasks. Viral and treatment effects in OF and LD were similar to EPM in stress naïve and stress-exposed conditions in both males and females, with vDG DREADD-mediated inhibition (Gi-DREADD + CNO) increasing time and distance in the center of the OF and the light compartment of the LD (Supplemental Figs. 3a–d & 3 h-k). Across the OF, LD, and EPM we found no differences in total distance traveled within VEH and CORT mice (p > 0.05 for all) (Supplemental Figs. 3e–g & 3 l-n). CNO treatment had no effects in OF, LD, and EPM relative to VEH in Control (AAV8-CamKIIa-GFP) injected mice. Taken together, these data indicate that cell autonomous inhibition of DG GCs decreases avoidance behaviors and that DG GC stimulation increases avoidance only on a non-stress background (possibly due to a ceiling effect on the stress background).

### 3.4 Ventral DG DREADD-mediated inhibition converts FLX non-responders into responders

We next wanted to determine if DREADD-mediated inhibition of vDG GCs is sufficient to convert behavioral non-responders to FLX treatment into responders. To this end, we injected Gi-DREADD virus into the vDG of 6-week-old mice (n = 30 males; n = 30 females), exposed these mice to chronic stress (4 weeks of chronic CORT for males and 7 weeks of SIS for females), and then co-administered FLX for 3 weeks (Fig. 4a–b). Mice next underwent NSF to assess behavioral response status with NR (n = 12 males; n = 12 females) and R (n = 18 males; n = 18 females) emerging (Fig. 4c–d). We then randomly treated half the NR and R with VEH and the other half with CNO for 3 weeks, and then exposed the mice to a second NSF trial (Fig. 4a–b). In the second NSF, we found significant differences between the 4 groups (NR + VEH, NR + CNO, R + VEH, R + CNO) (males: x(3) = 9.377, p = 0.025; females: x(3) = 35.7, p < 0.0001) (Fig. 4c–d). Interestingly, CNO decreased latency to eat in NR relative to VEH-treated NR (males: p = 0.001, females: p = 0.0005) (Fig. 4c–d). Gi-DREADD activation did not change NSF latency to eat in R mice (males: p = 0.366, females: p = 0.489). Thus, chronic vDG inhibition with Gi-DREADDs converts non-responders to fluoxetine treatment into responders in both male and female mice exposed to disparate chronic stressors.

### 3.5 Ventral DG DREADD-mediated stimulation converts FLX responders into non-responders

Following a similar design, we next explored if chronic stimulation of the ventral DG would lead to conversion of responders into non-responders. All mice in this experiment (n = 18 males, n = 30 females) were injected with a Gq-DREADD virus into vDG, exposed to chronic stress (4 weeks of chronic CORT for males and 7 weeks of SIS for females), and then co-administered FLX for 3 weeks (Fig. 4a–b). Mice next underwent NSF to assess behavioral response status with NR (n = 8 males; n = 7 females) and R (n = 10 males; n = 23 females) emerging (Fig. 4e–f). We then randomly treated half the NR and R with VEH and the other half with CNO for 3 weeks, and then exposed the mice to a second NSF trial (Fig. 4e–f). In the second NSF, group differences were found between the 4 Gq-DREADD groups (NR + VEH, NR + CNO, R + VEH, R + CNO) (males: x(3) = 23.46, p < 0.001; females: x(3) = 38.6, p < 0.0001) (Fig. 4e–f). CNO-mediated activation of Gq-DREADDs in vDG increased latency to eat in R relative to VEH-treated R (males: p = 0.002, females: p < 0.0001). No differences in NSF latency to eat were found within the NR groups (males and females: p > 0.99). Taken together, these data demonstrate that chronic modulation of DG activity with DREADDs is sufficient to bidirectionally convert FLX responder status in both male and female mice exposed to disparate chronic stressors.

## 4 Discussion

Here we illustrate the importance of the DG in mediating the response to antidepressant treatment in both male and female mice. Behavioral response to FLX is associated with a decrease in DG GC activity with DREADD-mediated chronic inhibition of vDG mounting antidepressant-like effects in stressed mice. Furthermore, we demonstrate that behavioral non-responders to FLX can be converted into responders with DREADD-mediated vDG chronic inhibition. By contrast, behavioral FLX responders are converted into non-responders following chronic DREADD-mediated vDG stimulation. Taken together, these results further demonstrate that DG GC inhibition is a critical component of the behavioral response to FLX.

### 4.1 Behavioral response to FLX

Within the United States, 16% of the population will experience an episode of major depression in their lifetime (Smith 2014). Although commonly used treatments, such as SSRIs, are readily prescribed to reprieve depressed patients of their symptoms, only a subset of patients (~33%) achieve remission with initial treatment (Trivedi et al., 2006). In both male and female rodents, we observed a bimodal distribution in stress + FLX groups (CORT + FLX and SIS + FLX). Across both chronic CORT and SIS paradigms, we illustrate that behavioral response to FLX (CORT + FLX-R and SIS + FLX-R) results in a consistent decrease in avoidance behaviors in EPM and FST immobility compared to non-responders (CORT + FLX-NR and SIS + FLX-NR) and stress controls (CORT + VEH and SIS + VEH). Given that females have historically been excluded from preclinical stress research, our results are the first to document this behavioral response status to FLX in female mice. Whether there are sex differences in SSRI efficacy in humans remains unclear despite decades of studies. Some studies have found SSRIs are more effective in women, while several others have reported no differences in efficacy between sexes (Sramek et al., 2016). Although we did not directly compare male and female mice, we observed similar responder and non-responder to FLX populations in both sexes.

Our preclinical data highlights a growing dilemma in modern psychiatry, with a large subset of the population non-responsive to first line pharmacotherapies. Psychiatrists try to address this issue by switching non-remitters to SSRIs to a second antidepressant. In the STAR*D trial, Rush and colleagues (2006) switched citalopram (SSRI) non-remitters to either bupropion (norepinephrine dopamine reuptake inhibitor), venlafaxine (serotonin norepinephrine reuptake inhibitor), or sertraline (SSRI) and measured their response to these second line antidepressants. However, these second line therapies only result in a ~33% remission rate despite these drugs acting on different monoaminergic neurotransmitters. These results highlight the need for a better understanding of the molecular and cellular action of antidepressants as well as novel approaches that address the heterogeneous nature of depression. Here we showed that individual differences in DG activity underlie antidepressant behavioral response status in mice.

### 4.2 Disparate chronic stressors in males and females

Arguably, one weakness of our study is that we used completely disparate chronic stressors in males and females. Corticosterone administration to males is a pharmacological stressor and exposure of females to social instability is a social (or psychological) stressor. Furthermore, females were exposed to seven weeks of social instability prior to FLX administration and males were only exposed to four weeks of CORT administration before starting FLX treatment. Therefore, we were not able to make any direct statistical comparisons between males and females for any measures. These types of comparisons would require identical stress experiences for both sexes.

However, males and females respond differently to identical stressors. For example, the CORT administration paradigm mimics activation of the hypothalamus-pituitary-adrenal (HPA) axis (David et al., 2009). Many years of preclinical research in rodents led to an association between HPA axis activation and maladaptive behaviors (de Kloet et al., 2005; McEwen 1999), and several human studies associated the antidepressant response with normalization of impairments in the HPA axis negative feedback (Greden et al., 1983; Heuser et al., 1996; Holsboer-Trachsler et al., 1991; Linkowski et al., 1987). All this work led to ill-fated development and clinical trials of putative antidepressants that targeted the HPA axis. Most likely, sex differences contributed to the failure of these drugs (Kokras et al., 2019). Female rodents were largely excluded from the preclinical studies and the putative antidepressants were not effective in women. Not surprisingly, we and others found that CORT administration is not an effective stressor in female rodents (Mekiri et al., 2017; Yohn et al., 2019).

A counterargument to the disparate stressors being a weakness is that two completely different methods carried out in different sexes leading to similar conclusions is a strength of this study. Here, we found that differences in DG activity during behavior correlate with antidepressant response status, and that direct modulation of DG activity can bidirectionally control antidepressant response status. Therefore, the fact that we reached similar conclusions with a pharmacological stressor in male mice and with a social stressor in female mice suggest that ventral DG activity may be a generalizable readout of the antidepressant response and that direct modulation of DG activity is a potential therapeutic avenue for treatment resistance. The next step will be to determine if there are differences in DG activity between human remitters and non-remitters to antidepressant treatment. Relatively recent advances in neuroimaging analyses should allow for hippocampal subfield specific measurements (Treadway et al., 2015).

### 4.3 Chronic inhibition of the DG mimics the behavioral response to antidepressants

DG inhibition appears to be a critical component of the behavioral response to SSRIs.

The behavioral response to chronic FLX is mediated by Gi-coupled 5-HT_1A_ heteroreceptors in vDG, and direct acute optogenetic inhibition of the ventral hippocampus suppresses maladaptive avoidance behaviors (Bagot et al., 2015; Samuels et al., 2015). Here, we used Gi-DREADDs to chronically inhibit vDG GCs. In non-stressed mice, vDG inhibition mimicked an antidepressant response in LD and EPM, and in stressed mice, vDG inhibition converted FLX non-responders into responders in NSF. By contrast, in the absence of stress, chronic vDG stimulation mimicked the effects of stress in LD, EPM, and NSF, while in stressed mice, vDG inhibition converted FLX responders to non-responders in NSF. These results were similar across male and female mice exposed to disparate chronic stressors. We did not see effects of vDG inhibition of unstressed mice in NSF, which was possibly due to a floor effect. Similarly, we did not see effects of vDG excitation in stressed mice that did not receive FLX due to a possible ceiling effect. Moreover, we show no behavioral differences between GFP + CNO and GFP + VEH mice, which controls for any behavioral effects of CNO being metabolized to clozapine (Gomez et al., 2017; Manvich et al., 2018).

Importantly, adult hippocampal neurogenesis in the DG is necessary for the behavioral response to SSRI treatment (David et al., 2009; Santarelli et al., 2003). DG abGCs, through inputs from lateral entorhinal cortex and connections to local GABAergic interneurons, are capable of inhibiting mature GCs (Anacker et al., 2018; Burghardt et al., 2012; Drew et al., 2016; Ikrar et al., 2013; Lacefield et al., 2012; Luna et al., 2019). We investigated adult hippocampal neurogenesis in FLX responders and non-responders (Supplemental Fig. 5), and found that male responders to CORT + FLX and female responders to SIS + FLX showed increased numbers of dentate gyrus Ki67+, Dcx+, and Dcx + cells with tertiary dendrites, as well as increased maturation indices, relative to CORT or SIS mice treated with vehicle. By contrast, the effects of FLX on neurogenesis in non-responders were significantly attenuated relative to responders. Therefore, attenuated levels of adult neurogenesis in FLX non-responders relative to responders may have contributed to both the higher number of DG cFos + cells we observed in FLX non-responders following NSF exposure and the lack of behavioral response to FLX administration.

## 5 Conclusion

Overall, the behavioral response to FLX is related to a decrease in DG GCs activity in both male and female mice, irrespective of the chronic stressor. Additionally, we show that DREADD mediated inhibition of DG GCs mimics an antidepressant behavioral response and can convert FLX non-responders into responders. Delineating the role of the DG GCs in mediating the behavioral response to antidepressants could lead to the development of novel pharmacotherapies or neuroimaging biomarkers for determining response status to antidepressants.

## Sources of funding

This work was supported by 10.13039/100000025NIMH Grant MH112861 (BAS).

## CRediT authorship contribution statement

**Christine N. Yohn:** Supervision, Project administration, Writing - original draft, Writing - review & editing, Visualization, Methodology, Formal analysis, Investigation, Resources, Validation. **Andrew Dieterich:** Investigation, Validation. **Isabella Maita:** Investigation, Validation. **Allyson S. Bazer:** Writing - original draft, Investigation, Resources. **Emma Diethorn:** Investigation. **Debbie Ma:** Investigation. **Mark M. Gergues:** Investigation. **Pu Hu:** Investigation. **Benjamin A. Samuels:** Funding acquisition, Writing - review & editing, Writing - original draft, Conceptualization, Methodology, Formal analysis.
