# Supplementary material for: Behavioral response to fluoxetine in both female and male mice is modulated by dentate gyrus granule cell activity
Source: Neurobiol Stress. 2020 Oct 17;13:100257. doi: 10.1016/j.ynstr.2020.100257 (PMC7739193; doi:10.1016/j.ynstr.2020.100257)

Supplemental Results

Ventral Dentate Gyrus DREADD-mediated inhibition and DREADD-mediated stimulation on negative valence behaviors. To explore the impact of ventral Dentate Gyrus stimulation or inhibition, separate 2x3 ANOVAs (virus x treatment with CNO or VEH) with Bonferonni posthoc comparisons were conducted within each stress condition. Within stress naïve mice, we found significant interactions between CNO treatment and virus injected in OF center distance (males: $F(2, 24)=3.791$, $p=0.0371$) and duration (males: $F(2, 24)=6.85$, $p=0.0044$) in males, but not females (Sup. Figures 3a-b & 3 left). In males, VEH Gq-DREADD+CNO mice traveled more distance in the OF center than VEH Gq-DREADD+VEH (distance: $p=0.0078$). Additionally, male VEH Gi-DREADD+CNO mice spent more time in the OF center than Gi-DREADD+VEH ($p=0.0022$), Gq-DREADD+CNO($p=0.0003$), and GFP+CNO ($p=0.0014$) mice (Sup. Figure 3b left). Within chronic stress-exposed mice, we also found significant interactions between CNO treatment and virus injected in OF center distance (males: $F(2, 24)=4.369$, $p=0.024$; females $F(2,30)=18.79$,$p<0.0001$) and duration (males: F $F(2, 24)=2.97$, $p=0.07$; females: $F(2,30)=12.32$,$p=0.0001$ (Figures 3d-e & 3h-i right). CORT Gi DREADD+CNO male mice traveled more in OF center than CORT Gi-DREADD+VEH (distance: $p=0.0064$; time: $p=0.0036$), CORT Gq-DREADD+CNO (distance: $p=0.0001$; time: $p=0.0027$) (Sup. Figure 3a & 3bright). SIS Gi DREADD+CNO female mice traveled more and spent more time in the OF center than SIS Gi-DREADD+VEH (distance: $p<0.0001$; time: $p<0.0001$), SIS Gq DREADD+CNO (distance: $p<0.0001$; time: $p<0.0001$), and SIS GFP+CNO (distance: $p=0.0007$; time: $p<0.0001$) (Sup. Figure 3h & I right)

Next, in the LD we found significant interactions between CNO treatment and virus injected within stress naïve mice in LD distance traveled (males: $F(2, 24)=4.883$, $p=0.016$; females: $F(2, 30)=6.955$,$p=0.0033$) and time spent in the light (males: $F(2, 24)= 11.44$, $p=0.0003$; females: ($F(2,30)=9.5$, $p=0.0006$). In males, VEH Gq-DREADD+CNO traveled and spent less time in the light than Gi-DREADD+CNO (distance: $p=0.0011$; time: $p<0.0001$) and GFP+CNO (distance: $p=0.0019$; time: $p=0.0037$). Additionally, VEH Gi-DREADD+CNO male mice spent more time in the light than Gi-DREADD+VEH ($p<0.0001$) (Sup. Figure 3c left). Within females, CNTRL Gi-DREADD+CNO traveled more and spent more time in the light than Gq-DREADD+CNO (distance: $p=0.0006$; time: CNO $p<0.0001$), Gi-DREADD+VEH (time: $p<0.0001$), and GFP+CNO (time: $p=0.0008$) (Sup. Figure 3j & 3k left). CNTRL Gq-DREADD+CNO females traveled less in the light than GFP+CNO ($p=0.0036$) and Gq-DREADD+VEH ($p =0.0018$) (Sup. Figure 3j left). Within chronic-stress exposed mice, we also found significant interactions between CNO treatment and virus injected in LD light distance (male: $F(2, 24)= 13.04$, $p=0.0001$; female: $F(1,30)=1.147$,$p=0.0049$) and time (male: $F(2, 24)=26.57$, $p<0.0001$; females: $F(2,30)=43.69$,$p<0.0001$). CORT Gi-DREADD+CNO traveled and spent more time in the light than Gi-DREADD+VEH (distance: $p<0.0001$; time: $p<0.0001$), Gq-DREADD+CNO (distance: $p<0.0001$; time: $p<0.0001$), and GFP+CNO (distance: $p=0.0004$; time: $p<0.0001$) (Sup. Figure 3c & 3d right). CORT Gq-DREADD+CNO spent less time in the light of the LD than GFP+CNO ($p=0.0037$) (Sup. Figure 3d right). SIS Gi-DREADD+CNO traveled more and spent more time in the light than SIS Gi-DREADD+VEH (distance: $p<0.0001$; time: $p<0.0001$), SIS Gq-DREADD+CNO (distance: $p<0.0001$; time: $p<0.0001$), and SIS GFP+CNO (distance: $p=0.0222$; time: $p<0.0001$) (Sup. Figure 3l & 3k right). SIS Gq-DREADD+CNO spent less time in the light than SIS GFP+CNO ($p=0.0025$) (Sup. Figure 3k right). Total distance traveled in OF, LD, and EPM was not impacted by changes in ventral DG activity in males and females (Sup. Figure 3e-g & 3l-n).

Behavioral Responders to Fluoxetine have Increases in Hippocampal Neurogenesis.

To assess the role of the DG in facilitating the behavioral response to antidepressants, we treated separate cohorts of male and female mice with 4 weeks of CORT or VEH administration and SIS or CNTRL exposure, respectively. We then administered either VEH or FLX for 3 additional weeks (timelines: Figures 2a & 2g). Following chronic FLX or VEH treatment, mice underwent NSF and were then sacrificed 40 minutes post-NSF to investigate expression of the immediate early gene cFos in DG (Figure 2) and markers of hippocampal neurogenesis (Sup. Figure 5). We collected and stained 1 out of every 6 sections containing the DG (total of 12 sections counted) to assess DG Ki67 (cellular proliferation, 1:500, Abcam, ab16667) and DCX expression (1:500; doublecortin, ThermoFisher). To differentiate between mature and immature neurons we counted total number of DCX cells, total number of DCX cells with tertiary dendrites, and then calculated a maturation index ratio. Two-way ANOVAs revealed FLX treatment (males: $F(1,36)=26.76$, $p<0.0001$; females: $F(1,36)=15.42$, $p=0.0004$) and chronic stress in males (CORT in males: $F(1,36)= 13.06$ $p=0.009$), but not in females $F(1,36)=1.52$ $p=0.22$), significantly impacted Ki67 expression in the DG (Sup. Figures 5a & 5e left). To investigate differences in Ki67 expression between CORT/SIS-only treated mice, NSF-defined CORT/SIS+FLX responders, and non-responders, we used one-way ANOVAs, which showed CORT/SIS+FLX-R mice have more DG Ki67+ cells than CORT/SIS+VEH (males and females: $p<0.001$) and CORT/SIS+FLX-NR mice (males and females: $p<0.001$) (Sup. Figure 5a & 5e right).

Next, separate two-way ANOVAs showed FLX treatment (males: $F(1,36)=10.92$, $p=0.0022$; females: ($F(1,36)=15.49$, $p=0.0004$) increased, while chronic stress exposure (CORT in males: $F(1,36)= 7.19$, $p=0.011$; SIS in females: $F(1,36)= 37.91$, $p<0.0001$) decreased DG DCX expression. Similar to Ki67, differences in DCX expression were further explored with one-way ANOVAs, which showed CORT/SIS+FLX-R mice have more DG Ki67+ cells than CORT/SIS+VEH (males and females: $p<0.001$) and CORT/SIS+FLX-NR mice (males and females: $p<0.001$) (Sup. Figure 5b & 5f right). Additionally, two-way ANOVAs revealed FLX treatment increased (males: $F(1,36)=21.86$, $p<0.0001$; females: ($F(1,36)=13.88$, $p=0.0007$), while chronic stress exposure (CORT in males: $F(1,36)= 3.11$, $p=0.0086$; SIS in females: $F(1,36)= 8.57$, $p=0.036$) decreased arborization of DG DCX+ cells (Sup. Figure 5c & 5g left). Specifically, CORT/SIS+FLX-R mice had more DCX+ cells with tertiary dendrites than CORT/SIS+VEH (males and females: $p<0.001$) and CORT/SIS+FLX-NR mice (males and females: $p<0.001$) (Sup. Figure 5c & 5g right). Lastly, two-way ANOVAs illustrated that the maturation index is increased by chronic FLX treatment (males: $F(1,36)=11.69$, $p=0.0016$, females: $F(1,36)=5.72$, $p=0.021$) and decreased by chronic stress (males: $F(1,36)=13.5$, $p=0.0008$, females: $F(1,36)= 8.71$, $p=0.042$) (Sup. Figure 5d & 5h left). Moreover, one-way ANOVAs revealed CORT/SIS+FLX-R mice had higher maturation indexes than CORT/SIS+VEH (males and females: $p<0.001$) and CORT/SIS+FLX-NR mice (males and females: $p<0.001$) (Sup. Figure 5d & 5h right).

Supplemental Figures and Legends

Supplemental Figure 1. NSF home cage latency to feed. (a & c) Home cage latency to feed for NSF related to characterizing responders and non-responders to FLX in males (a) and females (c). (b & d) Home cage latency to feed for cFos experiment males (b) and females (d). NSF latency to feed sample size (a) males: VEH+VEH = 15, VEH+FLX = 10, CORT+VEH=15, CORT+FLX = 31; (c) females: CNTRL+VEH = 10, CNTRL+FLX = 10, SIS+VEH=12, SIS+FLX = 21 (SIS+FLX-R= 15, SIS+FLX-NR=6. Sample sizes for cFos (b) males: sample sizes males: VEH+VEH = 5, VEH+FLX = 5, CORT+VEH=10, CORT+FLX = 20 (CORT+FLX-R= 10, CORT+FLX-NR=10); (d) females : CNTRL+VEH = 5, CNTRL+FLX = 5, SIS+VEH=10, SIS+FLX = 20 (SIS+FLX-R= 10, SIS+FLX-NR=10)

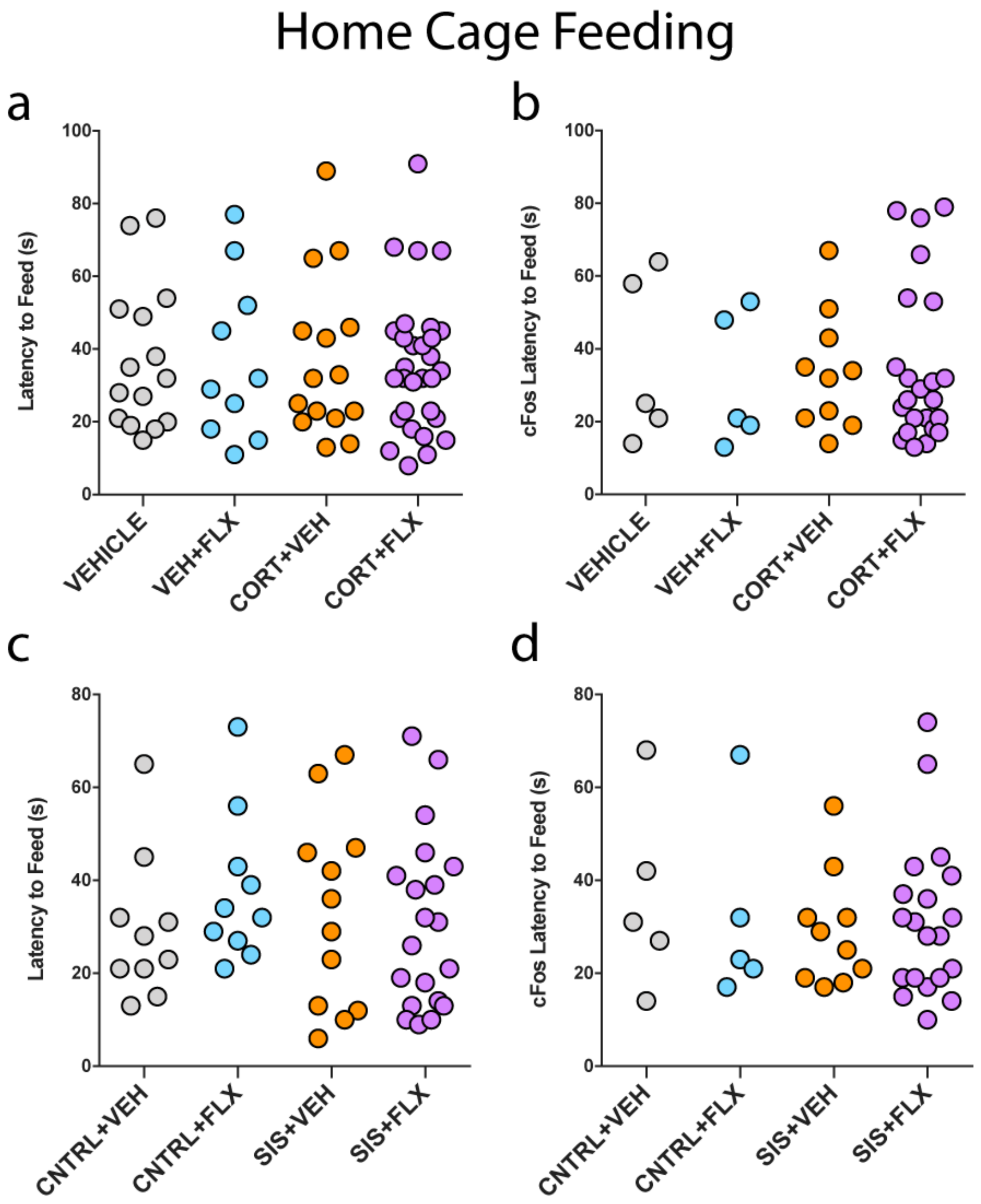

Supplemental Figure 2. DREADD mediated vDG inhibition or stimulation impacts vDG activity and does not impact control behavioral measures. (a) Gi-DREADD 4x images virus (mCherry), nuclear stain (DAPI), cFos expression (FITC). (b) Higher magnification of Gi-DREADD images (40x) used to count vDG cFos+ cells. (c & e) Male and female cFos counts to confirm CNO activation of DREADD virus. (d &f) Optical intensity to control for viral expression. Sample sizes males: all groups n=5; females: all groups n=6.

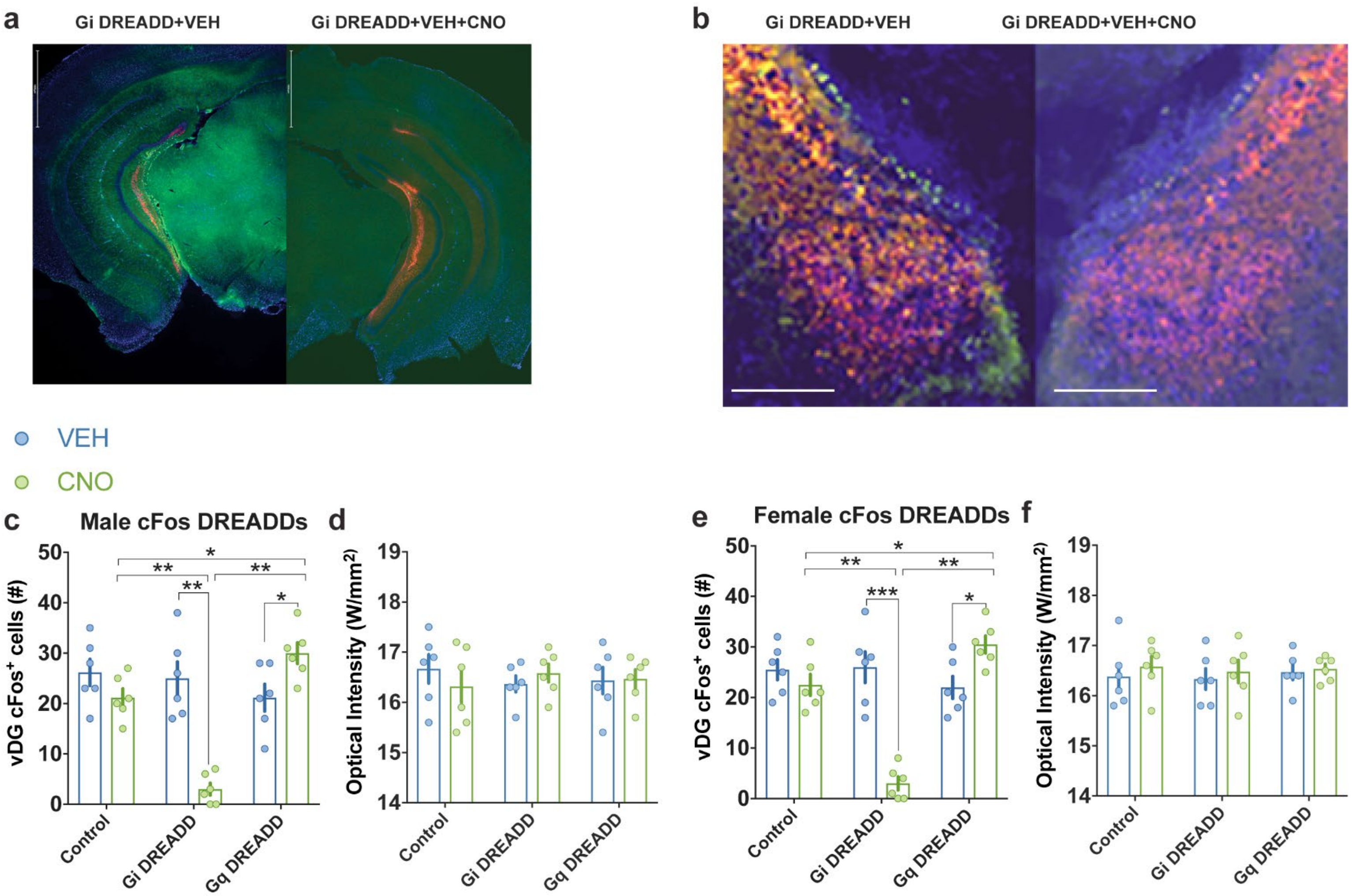

Supplemental Figure 3. DREADD mediated inhibition of the ventral Dentate Gyrus results in a decrease in negative valence behaviors in males (a-d) and females (h-k). Modulation of ventral Dentate Gyrus cellular activity does not impact total exploratory behavior within the different behavioral texts (males: e-g; females: l-n). Sample sizes males: all groups n=5; females: all groups n=6.

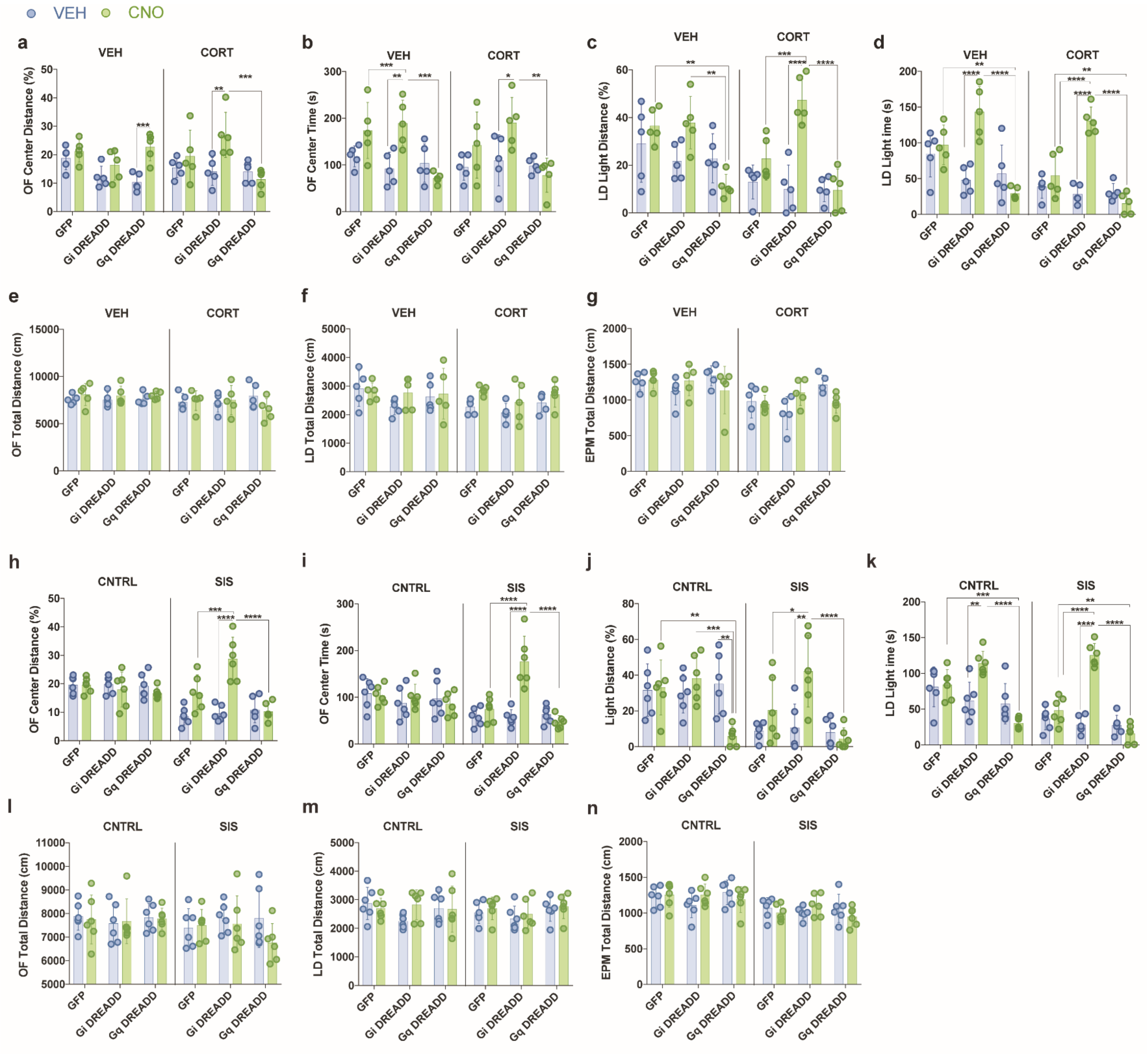

Supplemental Figure 4. (a & d) DREADD-mediated impact on home cage latency to feed following exposure to NSF. (b, c, e, & f) NSF representation survival curves for each stress group, showing percent that timed out (mice did not feed). Sample sizes males: all groups n=5; females: all groups n=6.

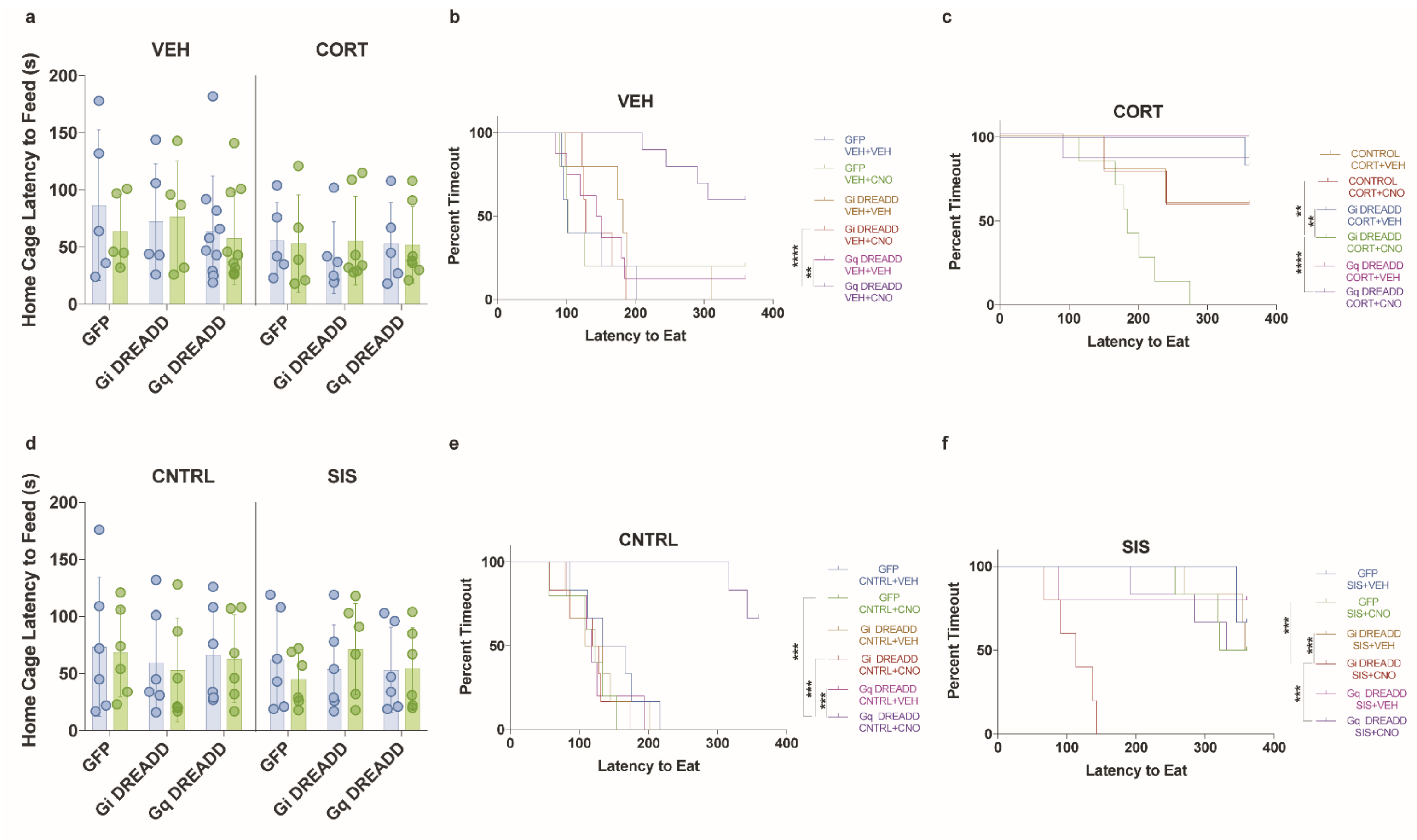

Supplemental Figure 5. Chronic fluoxetine increases hippocampal neurogenesis in male (a-d) and female (e-h) behavioral responders. Effects of fluoxetine (left panels) were observed on markers used to assess hippocampal neurogenesis Ki67 (cell proliferation; a & e) and DCX (cellular differentiation and maturation; b-c & f-g) as well as total maturation index (d & h). Assessing differences within the stress+fluoxetine group (right panels), behavioral male and female responders had increases in all neurogenesis markers (males: a-c; females: e-g) and maturation index (males: d; females: f) compared to stress controls and behavioral non-responders. (sample sizes males: VEH+VEH = 5, VEH+FLX = 5, CORT+VEH=10, CORT+FLX = 20 (CORT+FLX-R= 10, CORT+FLX-NR=10); females: CNTRL+VEH = 5, CNTRL+FLX = 5, SIS+VEH=10, SIS+FLX = 20 (SIS+FLX-R= 10, SIS+FLX-NR=10).

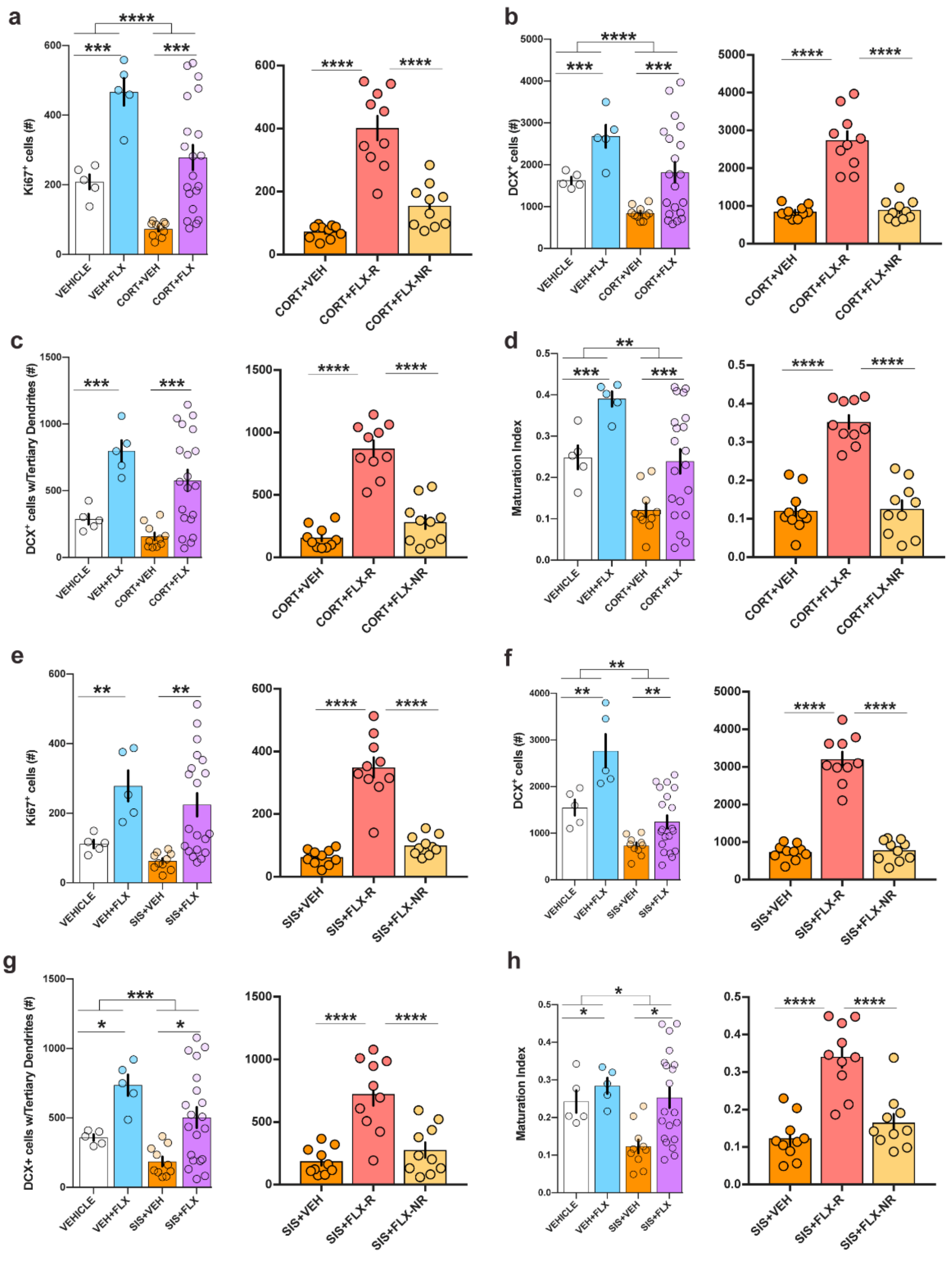

Supplement: Multimedia component 1 [file mmc1.pdf]
